# Data exclusivity exceptions and compulsory licensing to promote generic medicines in the European Union: *A proposal for greater coherence in European pharmaceutical legislation*

**DOI:** 10.1186/s40545-017-0107-9

**Published:** 2017-06-28

**Authors:** Ellen F. M. ‘t Hoen, Pascale Boulet, Brook K. Baker

**Affiliations:** 1 University of Groningen, University Medical Center Groningen, Global Health Unit, Hanzeplein 1, 9713 GZ Groningen, The Netherlands; 2 Consultant Medicines Law & Policy, 105 route de Lossiège, 74130 Contamine sur Arve, France; 3 Northeastern University School of Law, 400 Huntington Ave., Boston, MA 02115 USA; 4 University of KwaZulu Natal, Durban, South Africa

**Keywords:** Medicines pricing, Intellectual property, Access to medicines, Patents, Compulsory licensing, European Union, Data exclusivity, Market exclusivity

## Abstract

The challenge of providing access to high-priced patented medicines is a global problem affecting all countries. A decade and a half ago the use of flexibilities contained in the World Trade Organization Agreement on Trade Related Aspects of Intellectual Property Rights, in particular compulsory licensing, was seen as a mechanism to respond to high-price medicines for the treatment of HIV/AIDS in low- and middle-income countries. Today a number of upper-income European Union (EU) Member States are contemplating the use of compulsory licensing in their efforts to reduce expenditure on pharmaceutical products. EU regulation of clinical test data protection and the granting of market exclusivity interfere with the effective use of compulsory licensing by EU Member States and can even prevent access to off-patent medicines because they prohibit registration of generic equivalents.

EU pharmaceutical legislation should be amended to allow waivers to data and market exclusivity in cases of public health need and when a compulsory or government use license has been issued. Such an amendment can be modelled after existing waivers in the *EU Regulation on compulsory licensing of patents for the manufacture of pharmaceutical products for export to countries with public health problems outside the EU.* Allowing a public health/compulsory license exception to data and market exclusivity would bring greater coherence between EC regulation of medicinal products and national provisions on compulsory licensing and ensure that Member States can take measures to protect public health and promote access to medicines for all.

## Protection of clinical test data, and exceptions to data exclusivity to allow registration of generic medicines

This paper addresses the issue of clinical test data regulation in the European Union, which currently prohibits the use of the originator’s pre-clinical and clinical test data in the processing of a marketing authorisation for a generic medicine for a period of eight years. This is called the data exclusivity. After the eight years have passed the regulatory authorities can process the generic company’s application for marketing authorisation but the product may not be put on the market until ten years have passed after the initial marketing authorisation of the originator product. This is called market exclusivity. Under certain circumstances, an additional one year of market exclusivity may be obtained, for example when the originator company is granted a marketing authorisation for a significant new indication. This system of data and market exclusivity is also known as the 8 + 2 + 1 rule. The EU pharmaceutical legislation has no exception to this rule, which means that EU countries cannot register a generic product during the data/market exclusivity period, even when the medicine is needed for compelling public health reasons or emergencies or when a compulsory or government use license has been issued on a medicine patent. This paper will make recommendations for necessary changes to the EU pharmaceutical legislation to enable individual EU Member states to grant public health exceptions to data/market exclusivity and to make effective use of compulsory licences.

## Data exclusivity and TRIPS

Data exclusivity and market exclusivity are not requirements of international intellectual property law. While Article 39.3 of the TRIPS Agreement requires governments to *protect* undisclosed test data submitted for the registration of new chemical entities *against unfair commercial use*, it does not oblige countries to confer exclusive rights over data related to marketing approval to the originator company [1]. The scope of TRIPS Art. 39.3 is limited to the *protection* of undisclosed data required by a national authority as a condition for obtaining marketing approval for a medicine, which ‘utilize new chemical entities,’ provided that the generation of the data involved a considerable effort [2]. Article 39.3 of TRIPS therefore leaves ample flexibility for a data protection regime that allows the marketing authorisation of generic medicines. It also leaves flexibility to deal with regimes, as in the EU, where TRIPS-plus data exclusivities are granted.

However, as discussed further below, the EU medicines legislation goes well beyond the requirements of the World Trade Organization (WTO) Trade Related Aspects of Intellectual Property Rights (TRIPS) Agreement Art.39.3 in granting exclusive rights that form an obstacle to the effective use of compulsory licensing by EU Member States regardless of the reasons for the licence and even in emergency situations.

In a 2006 letter to the European Generic Medicines Association, which was seeking clarification on whether data exclusivity would apply in case of an emergency compulsory licence for the flu medicine Tamiflu within the European Union, the European Commission acknowledged that the ‘Community pharmaceutical *acquis* does not currently contain any provision allowing a waiver of the rules on data exclusivity and marketing protection periods’ [3]. The European Commission, however, has yet to take any initiative to propose such a waiver in pharmaceutical legislation.

## Compulsory licensing for public health

The WTO TRIPS [4] includes provisions for compulsory licensing, a mechanism whereby a government grants third parties or itself the right to use a patent without the consent of the patent holder. When a government grants itself the right to make use of a patent, this is called ‘government use’, or ‘public non-commercial use’.^1^ Government use or public non-commercial use of a patent can be particularly useful in public procurement of medicines. A government may also authorise a third party to act on behalf of the government, for example, a medicines procurement agent, to perform certain acts that otherwise would have constituted a patent infringement. Payment of adequate remuneration – a reasonable royalty – to the patent holder is required when a compulsory licence is granted. In the case of government use, in case of a national emergency or other circumstances of extreme urgency, and in cases where a compulsory licence is issued to correct anti-competitive practices, there is no requirement to first seek a voluntary licence. 2 3

The government is free to determine the grounds for granting a compulsory licence. Some countries’ domestic law includes specific grounds for issuing a compulsory licence such as ‘high prices’ of medicines, or a ‘lack of access to medicines’. For example, French patent law authorises government use upon request by the minister of health when medicines are ‘only available to the public in insufficient quantity or quality or at abnormally high prices [5]’.

In 2001 the WTO Doha Declaration on the TRIPS Agreement and Public Health [6] provided a welcome clarification of the flexibilities [4]^4^ contained in the TRIPS Agreement for the purpose of public health and specifically to promote ‘access to medicines for all’ [7]. With the background of trade pressure on low- and middle-income countries that contemplated the use of compulsory licensing and other TRIPS-flexibilities, the Doha Declaration took away any doubts about the legality of such measures. Subsequently, low- and middle-income countries have used TRIPS flexibilities on a large scale to facilitate the supply of low-cost generic medicines used for the treatment of HIV [8].

More recently, interest in the usage of TRIPS flexibilities for a broader range of health products has been growing. The UN High Level Panel on Access to Medicines recommended the use of TRIPS-flexibilities and the implementation of legislation that facilitates the issuance of compulsory licences ‘*designed to effectuate quick, fair, predictable and implementable compulsory licenses for legitimate public health needs*’ [9]. The Lancet Commission on Essential Medicines Policies recommended that national patent legislation allow for easy deployment of TRIPS flexibilities, effective automatic licensing of essential medicines in the absence of voluntary agreements, and regulatory test data protection rules that provide the necessary flexibility to register products submitted by licensees [10]. These recommendations echo those from the Global Health Law Committee of the International Law Association [11]. The European Parliament has adopted a resolution on options for the EU for improving access to medicines, which includes the use of compulsory licensing by EU Member States [12].

## High medicines prices, compulsory licensing and data exclusivity in the European Union

A decade and a half ago, the use of TRIPS flexibilities and in particular compulsory licensing was seen primarily as a mechanism to respond to the HIV/AIDS crisis in low- and middle-income countries. Today, a number of EU Member States, including high-income countries, struggle to formulate an effective response to high-priced patented medicines. In the UK, the National Institute for Health and Care Excellence (NICE) has recommended against making the breast cancer medicine trastuzumab emtansine available through the National Health Service because of the high price, while recognising that the medicine is effective [13]. In Italy the government has authorised the importation of generic direct-acting antiviral medicines for the treatment of hepatitis C on an individual basis to increase access to these medicines [14]. In Switzerland, patients denied access to new hepatitis C treatment can receive reimbursement by some insurance companies when they source generic medicines for the treatment in India at a lower price [15].

Governments have signalled that they lack the negotiating power to obtain good results in price negotiations concerning patented products [16, 17], despite the fact that production-cost data show that medicines can often be made for a fraction of the price demanded by the originator company (Table 1). The Dutch ministers of health and international trade wrote in the Lancet about the challenges of negotiating with patent holders: “Patent and intellectual property exclusivities are the only cornerstone of the current model. Companies can ask the price they like. This will no longer do. We need to develop alternative business models” [18].

**Table 1:** Price of selected essential medicines and cost of production [52–54]

Medicine	Originator price intro US	Cost of production
Tuberculosis		
Bedaquiline	$ 30000 ( 6 month)	$ 48–101
Hepatitis C		
Sofosbuvir (SOF)	$ 84000 (12 week)	$68–136
SOF + ledipasvir	$ 95000 (12 weeks)	$ 193
Simeprevir	$ 66360 (12 weeks)	$130–270
Daclatasvir	$ 63000 (12 weeks)	$10–30
Cancer		
Imatinib	$ 30000–$100000 (1y)	$ 119–159
Trastuzumab	$54000 (1 y)	$ 242

Patients and medical professionals and in some cases health authorities in high-income countries have become more vocal in asking their governments to address the patent barriers to accessing lower-priced medicines and to invoke compulsory licensing [19–24].

Referencing the lawful production or importation of affordable generic medicines through the use of compulsory licences will strengthen the hand of governments in price negotiations and is an effective remedy if price negotiations fail to deliver the desired result. There are lessons from the past when government use was routine in the procurement of medicines for use in national health systems; for example, in the sixties and seventies in the UK, compulsory licensing had become common practice in government procurement by the NHS. Attempts by the industry to halt this practice at the time failed [25].

However, the ability of the EU to provide more affordable access to a patent-protected medicine through a compulsory licence may be hindered if the originator company’s product simultaneously benefits from data exclusivity. Data exclusivity refers to exclusive rights granted to the original manufacturer of a medicine over the use of test data required for the registration of the product. These exclusive rights are distinct from patent rights in that they are granted by the medicines regulatory authority^5^ in relation to safety and efficacy data submitted for the approval of originator medicines.

According to the EU Regulation for the authorisation and supervision of medicinal products [26], a generic medicine may only be authorised with reference to the originator’s registration file once eight years of data exclusivity has passed, and may only be placed on the market ten years after the initial marketing authorisation for the originator product has been granted. This marketing period may be extended to 11 years in cases of new indications that have a significant clinical benefit over existing indications.^6^ This means that a generic applicant cannot submit a market authorization based solely on bioequivalence data before the expiration of eight years and instead would have to provide self-generated pre-clinical and clinical trial test data, which generic companies typically do not do. Additionally, such clinical trials would mean an unnecessary duplication of studies [27] and raise ethical questions. In the EU data and market exclusivity applies to both small molecules and biologics.

At present, EU pharmaceutical legislation does not provide for exceptions to the eight to ten years data and market exclusivity. Even in cases of national emergency or other situation of urgency, there are no explicit waivers foreseen in EU law to address the need to authorise the marketing of a generic product before the aforementioned exclusivity periods expire. Even though issuing a compulsory licence to overcome patents blocking the use of a generic medicine is a matter of national law, regulatory requirements for EU-wide marketing authorisation, including data exclusivity, are a matter of European pharmaceutical legislation. These concurrent legal systems lack coherence, both with regards to the effective use of compulsory licensing by EU Member States and with respect to public interest exceptions to data exclusivity more broadly.

### Case of Romania

In 2016, the government of Romania contemplated issuing a compulsory licence for the hepatitis C medicine sofosbuvir, which, in Europe, was only available from the originator company at a price of around 50.000 euro for a 12-week treatment [28]. Since the registration of a generic version of sofosbuvir is not possible before the expiry of the data exclusivity in 2022 [29],^7^ Romania, like any other EU Member State, cannot give effect to a compulsory licence. Further, the EU market exclusivity for sofosbuvir expires at the earliest in 2024. The case of Romania reveals the obstacles to the effective use of compulsory licensing created by EU data exclusivity.

## Use of DE waivers in voluntary licences aimed at ensuring access to medicines

The need to provide data exclusivity waivers to ensure effective availability of generic medicines is often acknowledged in voluntary licences. For example, all Medicines Patent Pool [30] (MPP) licences include a data exclusivity waiver to facilitate regulatory approval of generic medicines manufactured by MPP’s licensees. These waivers are necessary to ensure that generic manufacturers that sign MPP licences are not prevented from registering their products in countries which are part of the licensed territory and which grant test data exclusivity. For instance, Guatemala is included in the territory of the MPP licences with ViiV Healthcare for paediatric formulations of dolutegravir (DTG) and for adult formulations of DTG and DTG/abacavir (ABC). The licences specifically state that:

ViiV shall provide any Sublicensee with NCE Exclusivity or other regulatory exclusivity waivers to the extent required by the applicable regulatory authorities in order to manufacture or sell Product in the Territory in accordance with the terms of the Sublicence. ViiV shall further provide to any Sublicensee such consents which it has the legal capacity to give as are necessary to enable such Sublicensee to perform its obligations [31].

As indicated in the patent database Medspal [32], the formulations of DTG 50mg and ABC/DTG/3TC 600/50/300 mg are protected by test data exclusivity in Guatemala until 11 November 2020 and 29 November 2021 respectively. However, MPP licensees will nevertheless be able to register and market generic versions of these formulations in Guatemala before the expiration of these rights, based on the waiver included in the MPP licence agreements.

Gilead has also included the following waiver of data exclusivity in its licence agreements for sofosbuvir:

Gilead agrees to provide Licensee with NCE Exclusivity, or other regulatory exclusivity, waivers as may be required by the applicable regulatory authorities in order to manufacture or sell Product in the Territory, provided such manufacture and sale by Licensee is compliant with the terms and conditions of this Agreement. Licensee agrees not to pursue or obtain regulatory exclusivity on any Product in any country within the Territory [33].

Even though Gilead obtained test data exclusivity for sofosbuvir 400 mg until 14 July 2021 in Guatemala, for instance, Gilead licensees cannot not be barred from registering and selling generic versions of SOF 400mg during this data exclusivity period in Guatemala, which is included in the licensed territory.

Governments, including in the EU, should be able to provide similar data exclusivity waivers.

## Data exclusivity waivers in national legislation in other jurisdictions

Some middle- and high-income countries, all of them members of the WTO and thus subject to the TRIPS Agreement, provide for explicit data exclusivity waivers in medicines regulations or in relation to the use of compulsory licences in patent laws, with a view to facilitating generic medicines registration and sales where necessary to protect public health.

For example, Section 5 of **Malaysia** 2011 Directive of Data Exclusivity [34], entitled Non-Application of Data Exclusivity, provides that

Nothing in the Data Exclusivity shall:

(i) apply to situations where compulsory licenses have been issued or the implementation of any other measures consistent with the need to protect public health and ensure access for all; or
(ii) prevent the Government from taking any necessary action to protect public health, national security, non-commercial public use, national emergency, public health crisis or other extremely urgent circumstances declared by the Government.

In **Chile**, Article 91 of Law 19.996, as amended in 2012 [35], provides that test data exclusivity shall not be applied as follows:

- Where, for reasons of public health, national security, public non-commercial use, national emergency or other circumstances of extreme urgency declared by the competent authority, it is justified to terminate the protection referred in Article 89 (e.g. on test data exclusivity).
- The pharmaceutical or agrochemical product is the subject of a compulsory license in conformity with the provisions of this law.

In **Colombia**, Article 4 of Decree 2085 of 2002 on data exclusivity provides that, ‘The protection referred to in this Decree does not apply in the following cases […] c) where necessary to protect the public, as qualified by the Ministry of Health’ [36].

## Other exceptions in the US: trade agreements and the New Trade Policy exception

The US data/marketing exclusivity rule on previously unapproved chemical entities (new small molecule medicines) is that there are five years of marketing exclusivity and that a generic may not apply for tentative marketing approval until after the fourth year and may do so only if the applicant certifies that the underlying patent is invalid or that the medicine will be non-infringing. Final or tentative approval is not available until at least the end of the fifth year [37].^8^ If the original period of exclusivity is extended with three years because of new clinical trial data involving a previously approved chemical entity, e.g., for a new use or new formulation, an application for tentative approval is possible any time during the three years [38]. For biologics, the effective marketing exclusivity term provided by the *Biologics Price Competition and Innovation Act* is 12 years from the date the reference product was first licensed; there is data exclusivity preventing even applications for tentative approval for the first four years [39].

As with the EU, there is no express exception in US law to data/marketing exclusivity on medicines or biologics. However, the 10 May 2007 New Trade Policy [40] in the US authorized an express public health exception to data/market exclusivity in the event of a compulsory licence or other public health need. Implementation flexibility to that effect was included in several US developing-country free-trade agreements (FTA), including FTAs with Colombia, Panama, and Peru:

For pharmaceutical products, Article 16.10.2(e)(i) provides an exception to the data exclusivity obligations for measures to protect public health in accordance with the Declaration on the TRIPS Agreement and Public Health (WT/MIN(01)/DEC/2) (the ‘Doha Declaration’). Thus, where a Party issues a compulsory licence in accordance with Article 31 of the TRIPS Agreement and the Doha Declaration, the data exclusivity obligations in Chapter Sixteen will not prevent the adoption or implementation of such a public health measure. In addition, in a case in which there is no patent on the pharmaceutical product, and, therefore, no need to issue a compulsory licence, the data exclusivity obligations in Chapter Sixteen will not prevent the adoption or implementation of such a measure [41].

The advantage of the US New Trade Policy approach is that it allows countries to disregard data/marketing exclusivity if they take measures to protect public health, regardless of whether a compulsory licence needs to be issued or not –depending on the patent status of the medicine in question.

## Existing EU legislation containing waivers or exceptions to data and market exclusivity

Waivers to data exclusivity and market exclusivity rules do exist in the *EU Regulation on compulsory licensing of patents for the manufacture of pharmaceutical products for export to countries with public health problems outside the EU* [42]. This regulation implements the WTO ‘August 30 2003 decision’, which provided a waiver to the TRIPS Article 31(f) requirement that production under a compulsory licence be predominantly for the domestic market. This restriction seriously hampered the use of compulsory licensing by countries that were dependent on the importation of medicines. The 30 August 2003 waiver recently became a permanent amendment of the TRIPS Agreement [43]. Article 18 of the EU Regulation addresses the situation in which the applicant for a compulsory licence for manufacture and export of a medicine outside the EU may use the scientific opinion procedure of the European Medicines Agency (EMA) [44, 45], or any similar national procedures, to assess quality, safety, and efficacy of medicines intended exclusively for markets outside the EU. It provides waivers to exclusivity rules necessary to obtain such opinions from the EMA or national authorities [42].^9^

Certain EU trade agreements establish, in regards to test data, that Member States may provide exceptions to exclusivity for reasons of public interest and for situations of national emergency or extreme urgency when it is necessary to allow access to certain data to third parties. Such a provision can be found, for instance, in Article 231(4) of the EU-Peru Agreement which reads: ‘*[t]he Parties may regulate exceptions for reasons of public interest, situations of national emergency or extreme urgency, when it is necessary to allow access to those data to third parties*.’ [46] In practice, this means that the EU and Peru, both party to this agreement, may provide and use data exclusivity waivers to ensure effective use of compulsory licence. The waiver may also be relevant for non-patented products that benefit from exclusivity in the market because of data exclusivity.

For example, in Peru, daclatasvir, used in the treatment of hepatitis C, is patented until 2027 and benefits from a five-year data exclusivity period set to expire in July 2019 [32]. If the relevant Peruvian authority issues a compulsory licence to authorise the supply of generic daclatasvir, the medicines agency can ignore the data exclusivity and provide the necessary marketing authorisation for the generic product.

Peruvian law includes a specific exception to data exclusivity that allows the registration authority to authorise third parties to use pharmaceutical test data for reasons of public health and situations of national emergency or extreme urgency. In addition, the legislation specifically authorises third parties to use or refer to the test data in their application to obtain registration in case of a compulsory licence [47].

The EU trade agreements create rights and obligations for all parties to the agreement, and therefore further strengthens the case for regulating explicit exceptions to data and market exclusivity in cases where a compulsory licence and/or other measures in the interest of public health are taken in the EU or by EU Member States.

## Recommendation for greater legislative coherence in the EU

The right of governments to grant compulsory licences, including for public non-commercial use, is acknowledged in international law, including in TRIPS. Effective use of such licences requires a waiver of data exclusivity for the approval and marketing of licensed generic medicines. However, such waivers do not exist under EU law and as a result, an entity authorized to make use of the patent to supply a generic medicine under a compulsory licence still might not be able to do so because it cannot obtain a marketing authorisation from the relevant medicine regulatory authority. This lack of legal coherence within the EU renders national compulsory licensing provisions useless with respect to EMA approved medicines protected by data exclusivity.

Some patent holders recognise the need to address the barrier to market entry that data exclusivity can create. They therefore include relevant waivers in voluntary licence agreements, to ensure that licensed rights can be used effectively by licensees. For example, all licence agreements of the Medicines Patent Pool contain such waivers. The need for data exclusivity waivers is also recognised in the US New Trade Policy of 2007 and certain bilateral trade agreements to which the EU is a party. Since a compulsory licence is a government remedy for the absence of a voluntary licence, the government should also be able to attach conditions to the licence including a waiver of data and market exclusivity [48]. Further, data and market exclusivity waivers should also be available in situations where a needed medicine is not protected by a patent but a public health concern requires its availability.

The EU regulation on the grant of compulsory licensing for export does contain waivers for data and market exclusivity. These waivers allow European regulatory authorities to review dossiers of such licensed generic medicines to address third countries needs for affordable medicines. A similar waiver should be available to facilitate effective use of compulsory licensing or other measures needed for the advancement of public health within the European Union.

There is an urgent need to bring coherence to EU law now that Member States are under pressure to seek ways to ensure the availability of new essential medicines without undue burden on their health budgets. EU health ministers have recognised that steps need to be taken to address the effects of highly priced patented medicines on their budgets [49]. Legal coherence can be achieved by inserting the following provision into the EU legal framework governing medicinal products for human use:

‘The protection periods set out in article 14 (11) of Regulation 726/2004 shall not apply in cases where it is necessary to allow access to and the use of pharmaceutical test data to register a generic of a reference medicinal product, which is or has been authorised under article 6 of Directive 2001/83/EC, for reasons of public interest including public health, in case of compulsory licensing of patents, including for public non-commercial use, and in situations of national emergency or extreme urgency’.

In cases other than compulsory licensing and public non-commercial use of patents where adequate remuneration for the patent holder is required, payment of an adequate remuneration for the use of test data to the holder of the marketing authorisation of the reference medicinal product could be required. The adequacy of the remuneration could be determined based on an audited disclosure of direct drug development expenditure by the originator [50]. Alternatively, the royalty guidelines for non-voluntary use of a patent on medical technologies published by the UNDP and WHO could provide guidance for setting a remuneration rate [51].

## Conclusion

Amending EU legislation to introduce waivers of data and market exclusivity requirements will ensure that European patients can benefit from flexibilities in patent law and that data and market exclusivities do not undermine EU Member States’ ability to take measures needed to protect and promote public health. The proposed amendment would bring greater coherence between European regulation of medicinal products and national provisions on compulsory licensing in EU member States. The ability to effectively apply compulsory licensing will also strengthen the position of EU Member States in price negotiations with pharmaceutical companies. When such negotiations do not bring a satisfactory result, Member States can resort to compulsory licensing and produce or import lower-priced products without the consent of the patent owner.

## Abbreviations

3TC: Lamivudine
ABC: Abacavir
DTG: Dolutegravir
EMA: European Medicines Agency
EU: European Union
FTA: Free-trade agreement
HIV/AIDS: Human Immunodeficiency Virus/Acquired Immune Deficiency Syndrome
MPP: Medicines Patent Pool
NCE: New Chemical Entity
NHS: National Health Service
SOF: Sofosbuvir
TRIPS: Agreement on Trade Related Aspects of Intellectual Property Rights
UK: United Kingdom
UN: United Nations
UNDP: United Nations Development Program
US: United States
WTO: World Trade Organization

## Footnotes

1 This paper will further use the term ‘compulsory license’ to refer to both compulsory licenses and government use or public non-commercial use of a patent.

2 The term ‘voluntary license’ is used to refer to situations where the originator manufacturer agrees to authorise another party to produce and supply an otherwise patent-protected product.

3 Countries are free to determine what constitutes a national emergency or other circumstances of extreme urgency and what constitutes a competition violation. A national emergency or other circumstances of extreme urgency are not preconditions for issuing a compulsory licence, though industry and rich country sometimes suggest otherwise. Such situations merely makes the compulsory licence process easier as no prior negotiations to attempt to seek a voluntary licence are needed.

4 The term ‘flexibilities’ is used to describe limitations and exceptions to exclusive rights that countries can deploy for reasons of public interest.

5 In the EU, this is the European Commission.

6 Article 14(11) of Regulation (EC) No 726/2004 (n 21) reads: ‘Without prejudice to the law on the protection of industrial and commercial property, medicinal products for human use which have been authorised in accordance with the provisions of this Regulation shall benefit from an eight-year period of data protection and a ten-year period of marketing protection, in which connection the latter period shall be extended to a maximum of 11 years if, during the first eight years of those ten years, the marketing authorisation holder obtains an authorisation for one or more new therapeutic indications which, during the scientific evaluation prior to their authorisation, are held to bring a significant clinical benefit in comparison with existing therapies.’

7 For an overview of the EU medicines marketing authorization procedure see: European Medicines Agency, ‘Marketing authorization’ (reference no. 25).

8 The industry gained another six-month period of data exclusivity as a reward for conducting pediatric trials on drugs via 21 U.S.C. § 355a(b).

9 Article 18(2) reads: If a request for any of the above procedures concerns a product which is a generic of a reference medicinal product which is or has been authorised under Article 6 of Directive 2001/83/EC, the protection periods set out in Article 14(11) of Regulation (EC) No 726/2004 and in Articles 10(1) and 10(5) of Directive 2001/83/EC shall not apply.
